# Qb technology – evaluating its use in adhd diagnosis within a child and adolescent mental health service

**DOI:** 10.1192/j.eurpsy.2021.600

**Published:** 2021-08-13

**Authors:** D. Manning, S. Olety

**Affiliations:** Heathy Young Minds, Pennine Care NHS Foundation Trust, Stalybridge, United Kingdom

**Keywords:** ADHD, CAMHS, QB Technology, school

## Abstract

**Introduction:**

Attention Deficit Hyperactivity Disorder (ADHD) is a neurodevelopment disorder characteristically compromising of three persistent symptoms; Inattention, hyperactivity and impulsivity. Within the Tameside and Glossop CCG continuous performance tests from the company QbTech are used to aid diagnosis.

**Objectives:**

The aim of this research is to evaluate the effectiveness of using both the QbCheck (triage tool) and QbTest (diagnostic tool) concordantly in the diagnostic pathway of ADHD in young people.

**Methods:**

20 Patients who had undergone both performance tests were identified and then five components evaluated in the QbCheck were then compared to the QbTest results.

**Results:**

In the five areas identified by both the QbTest and QbCheck up to 80% had the same outcomes in the two tests. However, in one area (hyperactivity) only 60% of QbChecks outcomes were replicated by the QbTest. The symptom of inattention most commonly correlated between the two tests. The average wait between tests was 9.8 months. 100% of those who scored on QB Check, received diagnosis of ADHD, suggesting high referrer specificity.

**Conclusions:**

QbCheck diagnostic outcomes are comparable to patients who have undergone both the QbCheck and QbTest, only having one of these continuous performance tests making up the ADHD diagnostic pathway could be cost and time saving in the pathway to diagnosis. As QbCheck can be completed within the child’s school this reduces the number of clinic appointments that need to be attended by patients and their families.

